# A Painful Grip: A Case of Gonococcal Arthritis Masquerading as Wrist Pain

**DOI:** 10.7759/cureus.93700

**Published:** 2025-10-02

**Authors:** Andrew Schleffer, Francesca Kroll, Gary Schwartz

**Affiliations:** 1 Department of Medical Education, Nova Southeastern University Dr. Kiran C. Patel College of Allopathic Medicine, Fort Lauderdale, USA; 2 Orthopaedic Surgery, Nova Southeastern University Dr. Kiran C. Patel College of Allopathic Medicine, Fort Lauderdale, USA

**Keywords:** arthrotomy, bone biopsy, diagnosis of osteomyelitis, disseminated gonococcal infection, extensor tenosynovitis, neisseria gonorrhoeae, wrist septic arthritis

## Abstract

Disseminated gonococcal infection (DGI) is a rare complication of *Neisseria gonorrhoeae (N. gonorrhoeae​​​​​​),* and it can present variably, often making the diagnosis challenging. While it typically affects the knee, wrist involvement, as seen in this case report, has also been reported. DGI most commonly presents either as an arthritis-dermatitis syndrome or as purulent arthritis resembling septic arthritis. The diagnosis can be challenging, particularly when symptoms are atypical and initial imaging is unrevealing. Blood cultures may be negative in many cases, and hence, providers must consider DGI even in the absence of microbiologic confirmation. The management depends on the severity and varies from patient to patient. We describe a case of gonococcal arthritis of the wrist presenting after a traumatic accident at work. This report highlights the importance of recognizing the variable presentations of DGI and the need for early, multidisciplinary management to ensure optimal outcomes.

## Introduction

*Neisseria gonorrhoeae (N. gonorrhoeae​​​​​​)* is a gram-negative diplococcus, and its infection is commonly acquired through sexual contact. It represents the second most common cause of sexually transmitted diseases in North America, and young adults are particularly vulnerable. While *N. gonorrhoeae* causes mucosal infections, it has also been shown to cause disseminated gonococcal infection (DGI). The prevalence of DGI is about 0.5-3.0% in patients infected with *N. gonorrhoeae* [1]. The two classical presentations of DGI are arthritis-dermatitis syndrome and suppurative arthritis. The former involves a triad of migratory polyarthralgia, tenosynovitis, and pustular skin lesions. The latter typically consists of purulent arthritis in one or more joints, usually the larger joints, and is not accompanied by skin pustules or inflammation of the tendon sheath. While arthritis-dermatitis syndrome is likely caused by deposition of immune complexes, septic arthritis is due to *N. gonorrhoeae *directly invading the joint [2].

Gonococcal arthritis frequently affects the knee, but can also affect other joints, including the hand, wrist, or ankle [2-4]. Prompt diagnosis is key, as a delay in the diagnosis of gonococcal arthritis can lead to significant morbidity. Additionally, it often mimics other arthritides, and antibiotic treatment can be further complicated by bacterial resistance. We present an unusual case of gonococcal arthritis of the wrist arising after a traumatic accident at work. This case is unique for several reasons, including the isolated wrist involvement of the infection and its onset after a traumatic event.

## Case presentation

A 55-year-old male presented to the orthopedic hand clinic with pain in the left wrist and hand. Three weeks prior, he had been at work when his left wrist had been caught in a furniture-wrapping machine. The patient’s wrist had been pulled rapidly from pronation to supination, causing immediate pain. The patient had been initially seen in the emergency department, during which X-rays had not demonstrated any pathology. The patient had been subsequently discharged. The persistence of pain, as well as the onset of numbness and tingling in the digits of the left hand, had prompted the patient to seek further treatment at the clinic. The patient had a past medical history significant for tuberculosis, which had been treated and resolved. He denied any history of diabetes, rheumatoid arthritis, gout, or ulcers. He admitted to occasional tobacco smoking and alcohol use. There were no known allergies, and the family history was unremarkable.

**Figure 1:**
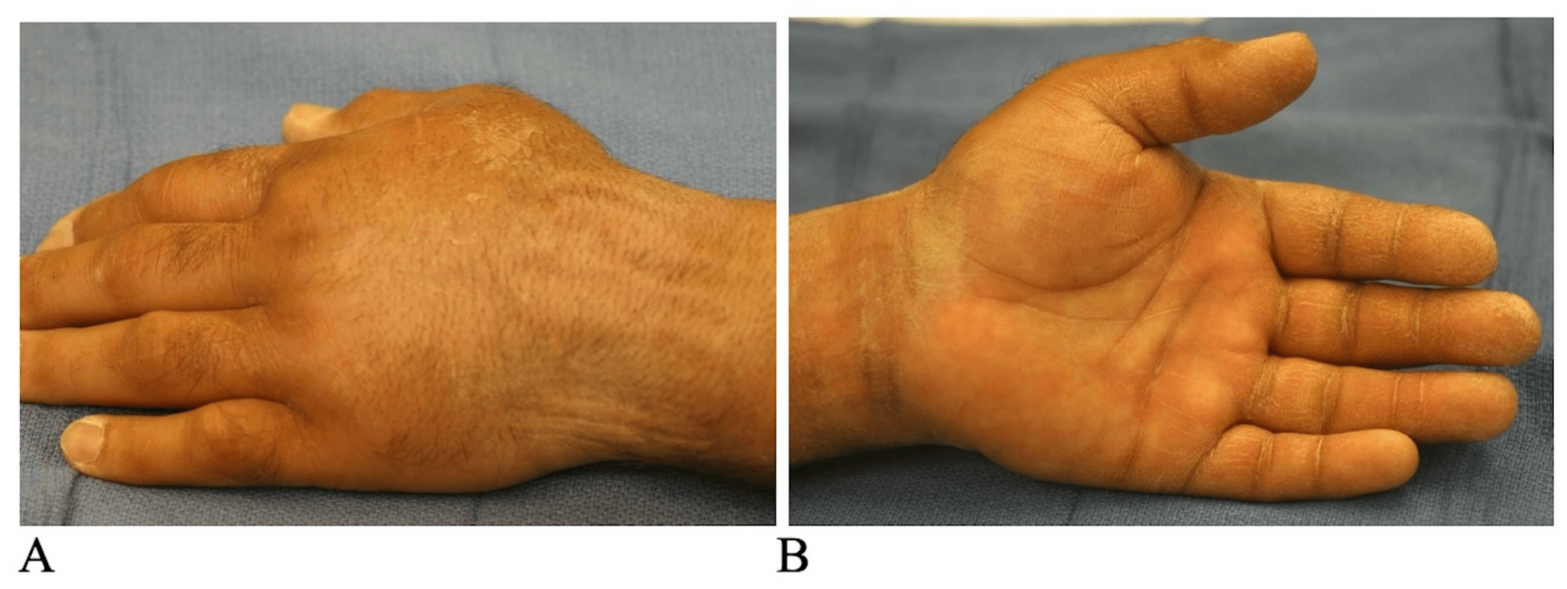
Initial preoperative clinical photographs The images demonstrate swelling on the dorsal (A) and volar (B) aspects of the left wrist and hand

A physical exam revealed tenderness on the volar and dorsal aspects of the left wrist with edema present (Figure 1). There was no fever, erythema, or any systemic signs. On examination, there were no vascular abnormalities. Capillary refill was less than two seconds. Two-point discrimination was 7 mm on all digits.

**Figure 2:**
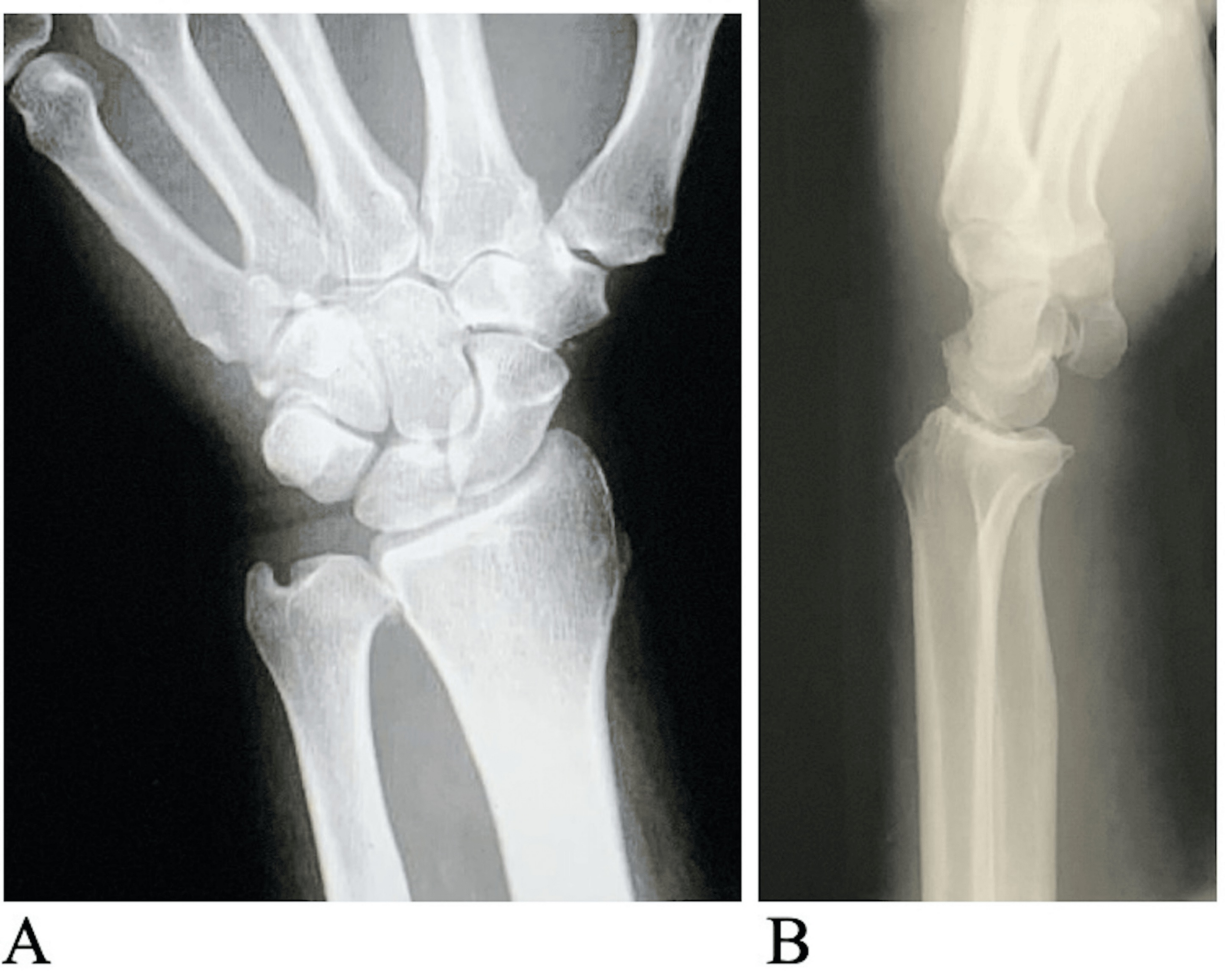
Initial preoperative anteroposterior (A) and lateral images (B) of the left wrist The images demonstrate no bony or joint abnormalities

There was a decreased range of motion in all digits and mild decreased sensation to light touch in the radial, ulnar, and median nerve distributions. Left wrist radiographs in the office revealed no obvious bony deformities (Figure 2). A volar splint was placed, and the patient was seen back in the office two weeks later.

**Figure 3:**
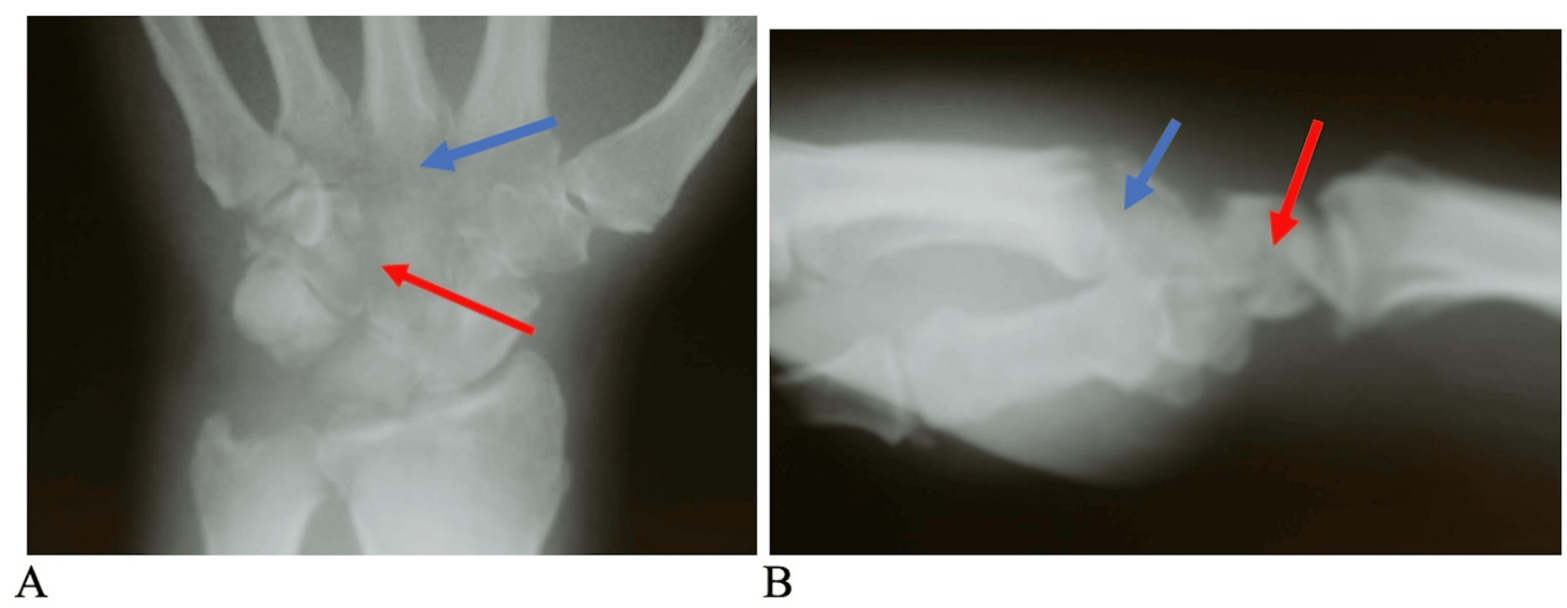
Preoperative anteroposterior (A) and lateral views (B) of the left wrist The images demonstrate lytic areas in multiple carpal bones (red arrows), the bases of the 2nd to 4th metacarpal bases (blue arrows), as well as destruction of the carpal-metacarpal and midcarpal joints

At the two-week follow-up visit, repeat radiographs revealed severe demineralization and osteopenia in all the carpal bones as well as the second, third, and fourth metacarpal bases (Figure 3). Given the dramatic change in the appearance of the radiographs over two weeks, it was felt that we were dealing with an aggressive infection. The initial laboratory results included a white blood count of 7,600 and a sedimentation rate of 66.

**Figure 4:**
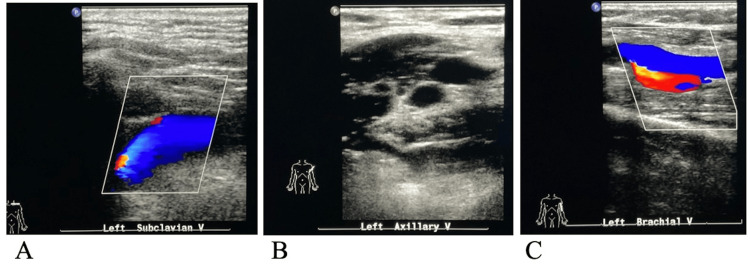
Left upper extremity venous Doppler exam The images do not demonstrate any evidence of deep venous thrombosis in the subclavian (A), axillary (B), or brachial (C) veins

A left upper extremity venous Doppler ultrasound did not reveal any evidence of thrombosis (Figure 4).

**Figure 5:**
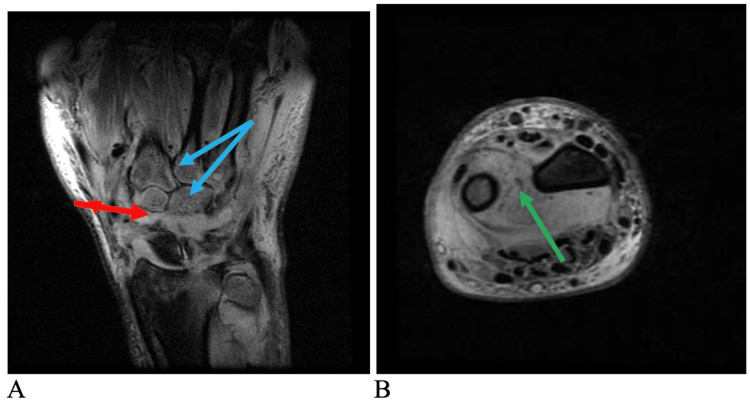
Preoperative MRI of the left wrist The images demonstrate abnormal signal intensity in the intercarpal region (red arrow). Bone marrow edema is identified in the proximal and distal carpal row as well as the 3rd to 5th metacarpal bases (blue arrows) (A). Synovitis is seen in the distal radio-ulnar joint and in the pronator quadratus (green arrow) (B) MRI: magnetic resonance imaging

An MRI revealed extensive marrow edema in the carpus, distal radius, and ulna, as well as myositis of the pronator quadratus extending proximally into the deep flexor and extensor compartments. Carpal synovitis as well as extensor tenosynovitis was noted (Figure 5).

**Figure 6:**
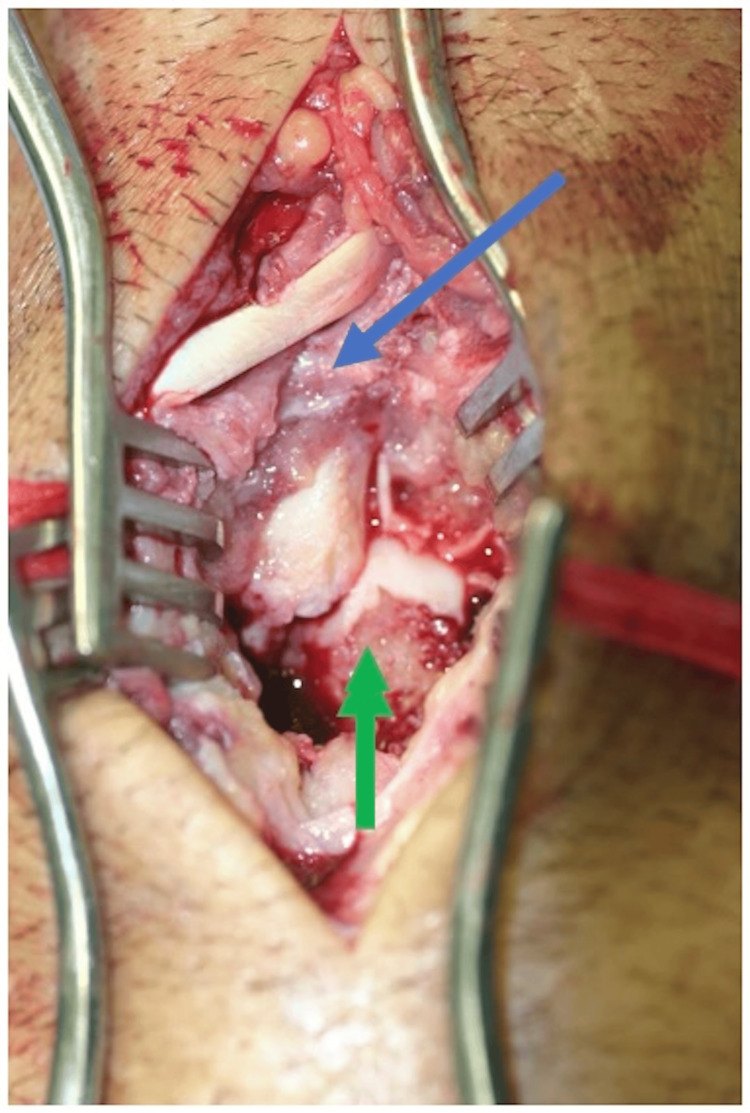
Intraoperative view of the dorsal aspect of the left wrist The image demonstrates granulation tissue (blue arrow) and bony erosions of multiple carpal bones (green arrow)

A diagnosis of septic arthritis of the left wrist with possible osteomyelitis was made, and the patient underwent aspiration of the dorsal aspect of the wrist. Purulent, bloody fluid was sent for Gram stain, aerobic/anaerobic, fungi, and atypical mycobacterium cultures. The patient returned the following day and underwent surgical incision, drainage, irrigation, debridement, arthrotomy, and bone biopsy of the left wrist. Intraoperative findings consisted of granulation tissue and bony erosions on multiple carpal bones (Figure 6).

Once the bone biopsy and cultures were taken, the patient was placed on intravenous Ancef, 2 grams IV every eight hours, until the intraoperative culture results were known. Ancef was chosen as the antibiotic due to the likelihood of the infection being caused by *Staphylococcus* bacteria. At 48 hours postoperatively, the cultures of the bone and joint subsequently demonstrated *N. gonorrhoeae*, sensitive to ciprofloxacin. At this time, as per infectious disease recommendations, the antibiotics were changed to oral ciprofloxacin, 500 mg every 12 hours. The oral antibiotics were continued for a period of six weeks. The patient underwent a course of occupational hand therapy for six months.

At six months postoperatively, the patient’s active wrist motion was 60 degrees of dorsiflexion, 30 degrees of volar flexion, 10 degrees of radial deviation, 25 degrees of ulnar deviation, 90 degrees of pronation, and 90 degrees of supination. The index through the small fingers came to within 2 cm from the distal palmar crease. The grip strength of the left hand measured on the Jamar dynamometer was 30 pounds compared to 85 pounds on the contralateral right side. The patient was discharged at this point. A Disability of the Arm, Shoulder, and Hand (DASH) score was not obtained by the hand therapist upon discharge of the patient. This questionnaire would be important to document the patient’s function and symptoms. Upon discharge, the sedimentation rate was within the normal range. 

## Discussion

*N. gonorrhoeae* causes a sexually transmitted infection that has seen a rise in incidence in recent years. According to the CDC, since 2019, cases of *N. gonorrhoeae infections* have consistently been above 600,000. Furthermore, over the past five years, there has been a +11.1% rise in cases [5]. This bacterium is typically spread through sexual contact, whether that be oral, vaginal, or anal in nature. Many of the presentations in infected persons are urogenital in nature, commonly manifesting as dysuria and/or vaginal discharge. Joints such as the knee or wrist are at increased risk of DGI. Common presentations of DGI typically follow one of two courses, the first of which is a triad consisting of tenosynovitis, skin lesions, and migratory polyarthritis (commonly referred to as an arthritis-dermatitis syndrome). The other presentation is known to be suppurative arthritis, similar to septic arthritis [2-4]. Some overlap of these forms of DGI has also been documented, but this was not the case in our patient.

Our patient demonstrated DGI leading to septic arthritis of the wrist with osteomyelitis following traumatic injury in the workplace. Although the knee is the most commonly affected joint in DGI, case reports involving the wrist have also been described [3,4]. The traumatic episode may have been indirectly involved in the etiology of the infection by making the wrist joint more vulnerable due to damage to the articular cartilage or soft tissue, allowing bacteria to localize to the wrist. The varying presentations of DGI mean that patients who do not present with “typical” symptoms are vulnerable to misdiagnosis and delay in proper treatment. Blood cultures are only found to be positive in less than a third of cases when patients are symptomatic [6]. Therefore, it is important for providers, including family practice physicians and orthopedists, to be aware of the varying presentations of DGI. Treatment of DGI may require serial monitoring with repeat X-rays and surgical intervention, because, as evidenced by this case, a singular negative radiographic image may not be sufficient. MRI or ultrasound may be utilized since they are more sensitive than radiographs in making the diagnosis.

The treatment of DGI varies from patient to patient and is based on the severity of symptoms and joint involvement. Often, intravenous antibiotics, most commonly ceftriaxone in combination with azithromycin, may be used. Ciprofloxacin may serve as an alternative in select cases, typically following initial IV therapy [4,7]. Current CDC guidelines recommend ceftriaxone-based therapy since there may be resistance to ciprofloxacin treatment. Previous CDC guidelines recommending dual therapy (ceftriaxone + azithromycin) for gonococcal infection were focused on slowing antibiotic resistance; however, the risks of dual therapy (potential deleterious effect on microbiome) outweigh the benefits. Therefore, ceftriaxone monotherapy with vigilance for treatment failure remains the gold standard in the treatment of DGI [8].

Surgical interventions, including arthrocentesis, incision and drainage, or arthrotomy, are often necessary when there is an inadequate response to antibiotics alone. Treatment duration ranges from one to four weeks, with some patients requiring transition to oral antibiotics and occupational therapy during recovery. Our patient underwent multiple surgical debridements/aspiration procedures, received IV cefazolin followed by oral ciprofloxacin, and was referred to occupational hand therapy for functional rehabilitation.

## Conclusions

This report highlights the fact that common signs and symptoms can have uncommon causes. The case was unique due to the isolated wrist presentation, the onset after a traumatic event, and antibiotic sensitivity. While *N. gonorrhoeae* is often considered in the context of its effect on mucosal surfaces, it is imperative to also remember that it can cause arthritis. Physicians need to be aware of the atypical presentation of gonococcal infections - when a patient comes in with an affected joint, DGI should not be overlooked. Serial imaging, including radiographs and, when necessary, an MRI, may be required to ensure that the diagnosis is not missed, resulting in delay of diagnosis and treatment. Clinicians of different backgrounds should keep DGI as a differential when evaluating any patients with atypical symptoms. This report serves as a reminder that typical diagnostic methods (such as blood cultures and radiographic imaging) may not initially point to DGI, but even in these situations, it should not be ruled out. It also demonstrates that trauma can indirectly be a contributing factor to the development of DGI, and it is often resistant to treatment with ciprofloxacin. Early detection, intervention, and treatment can significantly alter a patient’s clinical course and recovery.
